# YeastNet v3: a public database of data-specific and integrated functional gene networks for *Saccharomyces cerevisiae*

**DOI:** 10.1093/nar/gkt981

**Published:** 2013-10-26

**Authors:** Hanhae Kim, Junha Shin, Eiru Kim, Hyojin Kim, Sohyun Hwang, Jung Eun Shim, Insuk Lee

**Affiliations:** Department of Biotechnology, College of Life Science and Biotechnology, Yonsei University, Seoul, Korea

## Abstract

*Saccharomyces cerevisiae*, i.e. baker’s yeast, is a widely studied model organism in eukaryote genetics because of its simple protocols for genetic manipulation and phenotype profiling. The high abundance of publicly available data that has been generated through diverse ‘omics’ approaches has led to the use of yeast for many systems biology studies, including large-scale gene network modeling to better understand the molecular basis of the cellular phenotype. We have previously developed a genome-scale gene network for yeast, YeastNet v2, which has been used for various genetics and systems biology studies. Here, we present an updated version, YeastNet v3 (available at http://www.inetbio.org/yeastnet/), that significantly improves the prediction of gene–phenotype associations. The extended genome in YeastNet v3 covers up to 5818 genes (∼99% of the coding genome) wired by 362 512 functional links. YeastNet v3 provides a new web interface to run the tools for network-guided hypothesis generations. YeastNet v3 also provides edge information for all data-specific networks (∼2 million functional links) as well as the integrated networks. Therefore, users can construct alternative versions of the integrated network by applying their own data integration algorithm to the same data-specific links.

## INTRODUCTION

The complete mapping of gene-to-phenotype associations, which is a fundamental goal in the field of genetics, seems unconquerable due in part to the complex functional relationship among genes. However, the relationship among genes can be used to identify gene-to-phenotype associations based on the principle of guilt-by-association (1). Therefore, enormous efforts have been made to construct genome-scale gene networks to enable the effective prediction of novel gene functions and gene-to-phenotype associations in various organisms. This task requires the development of machine learning algorithms to infer and integrate functional links from a wide variety of biological data as well as efficient graph analysis algorithms to generate novel biological hypotheses from gene networks.

*Saccharomyces cerevisiae*, i.e. baker’s yeast, has been widely studied for two reasons. First, *S. cerevisiae* is a single-cell eukaryote in which many human cellular machineries have been conserved through evolution. Second, the genetic manipulation and phenotype profiling of *S. cerevisiae* are simple protocols to execute. Therefore, numerous high-throughput studies have been conducted, which have resulted in the generation of large data sets that have been deposited into public databases. Access to this abundant data, which has been derived from diverse experimental and informational technologies, provides a unique opportunity to develop methods in network construction and analysis. Over the past several years, we have used these data to develop genome-scale gene networks for yeast in YeastNet (2,3).

YeastNet v2 (3) significantly improved the original version of YeastNet (2) by removing the functional bias in the gold-standard links to enable functionally unbiased network training. YeastNet v2 included 5483 coding genes with 102 803 cofunctional links and showed high predictive power for independent pathway annotations and knockout (KO) phenotypes (4). YeastNet v2 proved to be useful for novel systems biology applications, including the network-based systematic reconstruction of the Gene Ontology of yeast genes (5), the prediction of the phenotypic effect of personal genome variations in yeast (6) and the prediction of epistasis (7). The success of YeastNet for these proof-of-concept novel systems biology studies has demonstrated the power of genome-scale gene networks in biological research. In the 5 years since the release of YeastNet v2, the availability of large-scale yeast data in public databases has continued to grow. The incorporation of these data into the existing network is expected to further improve the functionality of YeastNet.

Here, we present an updated version of YeastNet, YeastNet v3, that substantially improves phenotype prediction via several new approaches: (i) an extended and less-biased set of gold-standard cofunctional links for more efficient learning; (ii) the incorporation of a large number of additional large-scale experimental data; (iii) improved algorithms to infer functional associations from each data type; (iv) an improved web interface to serve various routs of novel hypothesis generation based on the principle of guilt-by-association; and (v) the availability of ∼2 million data-specific functional links that can be used to construct alternative integrated networks with user-defined integration methods.

## CONSTRUCTION OF YeastNet v3

The cofunctional links of nine different data types (CC, co-citation; CX, co-expression; DC, domain co-occurrence; GN, gene neighbor; GT, genetic interaction; HT, high-throughput protein–protein interaction; LC, literature curated protein–protein interaction; PG, phylogenetic profiles; TS, tertiary structure of protein) in YeastNet v3 are summarized in Table 1. YeastNet v3 includes 5818 genes (∼99% of the yeast coding genome) with 362 512 cofunctional links, which is ∼3.5 times the number of cofunctional links that were included in YeastNet v2. A total of 81 996 links (∼80% of YeastNet v2 links) were retained in the new network. The additional cofunctional links were inferred from new data and algorithms, which are described in the supplementary online methods.

**Table 1.** The cofunctional links of nine data types in YeastNet v3

Network description	Number of proteins (coverage of coding genome)	Number of functional associations
Co-citation (CC)	4355 (74%)	82 427
Co-expression (CX)	5730 (97%)	242 504
Domain co-occurrence (DC)	3679 (62%)	29 880
Genomic neighbor (GN)	1863 (32%)	29 475
Genetic interaction (GT)	4365 (74%)	149 498
High-throughput PPI^a^ (HT)	5487 (93%)	141 347
Literature curated PPI^a^ (LC)	5293 (90%)	54 421
Phylogenetic profiles (PG)	2463 (42%)	54 496
Tertiary structure of protein (TS)	1101 (19%)	3510
YeastNet v3	5818 (99%)	362 512

^a^PPI: protein–protein interaction.

## ASSESSMENT OF YeastNet v3 FOR IMPROVED PREDICTABILITY

### Assessment by pathway annotations

**Figure 1.**
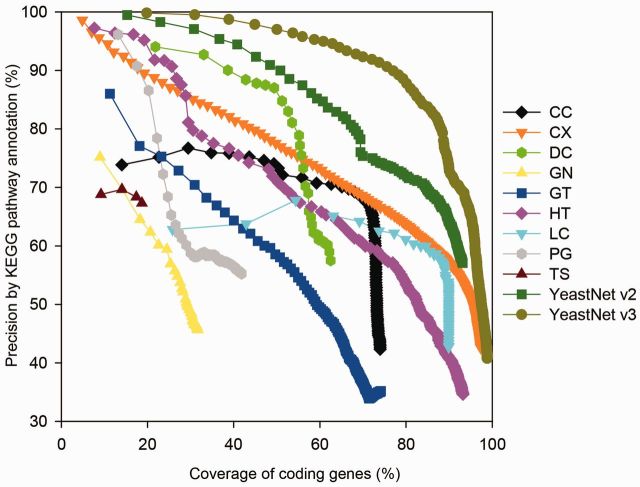
Precision-recall curves for YeastNet v3, YeastNet v2 and incorporated individual functional networks for nine distinct data types (CC, co-citation; CX, co-expression; DC, domain co-occurrence; GN, gene neighbor; GT, genetic interaction; HT, high-throughput protein–protein interaction; LC, literature curated protein–protein interaction; PG, phylogenetic profiles; TS, tertiary structure of protein). Precision was calculated from cofunctional links that were derived from the KEGG pathway annotation database, which were largely independent from the links in the network training set. Recall was measured as the percentage of coverage of the 5887 validated coding genes in the yeast genome. Gene pairs for each functional network were ranked by log likelihood scores from the benchmarking process, as described in the text. Precision and recall were calculated in a cumulative manner in which every consecutive 1000 gene pairs were binned (as indicated by each symbol). The plot shows that YeastNet v3 outperforms all other networks, including YeastNet v2.

To determine whether the rewired and additional functional links of YeastNet v3 improved the prediction of pathways and phenotypes in yeast, we assessed the predictive power of YeastNet v3 on different data sets. First, YeastNet v3 was assessed by pathway annotations in the Kyoto Encyclopedia of Genes and Genomes (KEGG) (8) database to test whether the measured accuracy of cofunctional links can be generalized. KEGG provides 279 327 positive and 1 385 073 negative gold-standard links to test. Only 10 064 links in the KEGG database (12.5%) overlap with the network construction in YeastNet v3, which guarantees that the validation set is independent from the training set. Precision-recall curves for the networks generated from the individual data types and integrated network are summarized in Figure 1. These curves show a substantial improvement in YeastNet v3 compared with the networks constructed by the individual data types as well as the previous version of YeastNet. Because the same benchmarking and integration methods were used in the current and previous versions of YeastNet, the improvements in the performance of YeastNet v3 are likely due to two important changes in the current version. First, the method of linkage discovery was changed for the phylogenetic profile similarity, genomic neighborhood and genetic interaction data types, and domain co-occurrence analysis was incorporated. Second, the amount of input data has grown significantly since the previous version release. The number of Medline abstracts that cite yeast genes grew from 39 135 to 46 111, and the number of reference genomes for the genome context methods grew from 149 to 2144. We also included six new high-throughput protein-protein interaction (PPI) data sets in YeastNet v3, and 1639 microarray samples were used in the current version of the network compared with the 500 microarray samples incorporated in the earlier version of the network. Therefore, both the algorithm improvements and the data expansion have enhanced the quality of the inferred molecular network.

### Assessment by quantitative phenotype profile data

**Figure 2.**
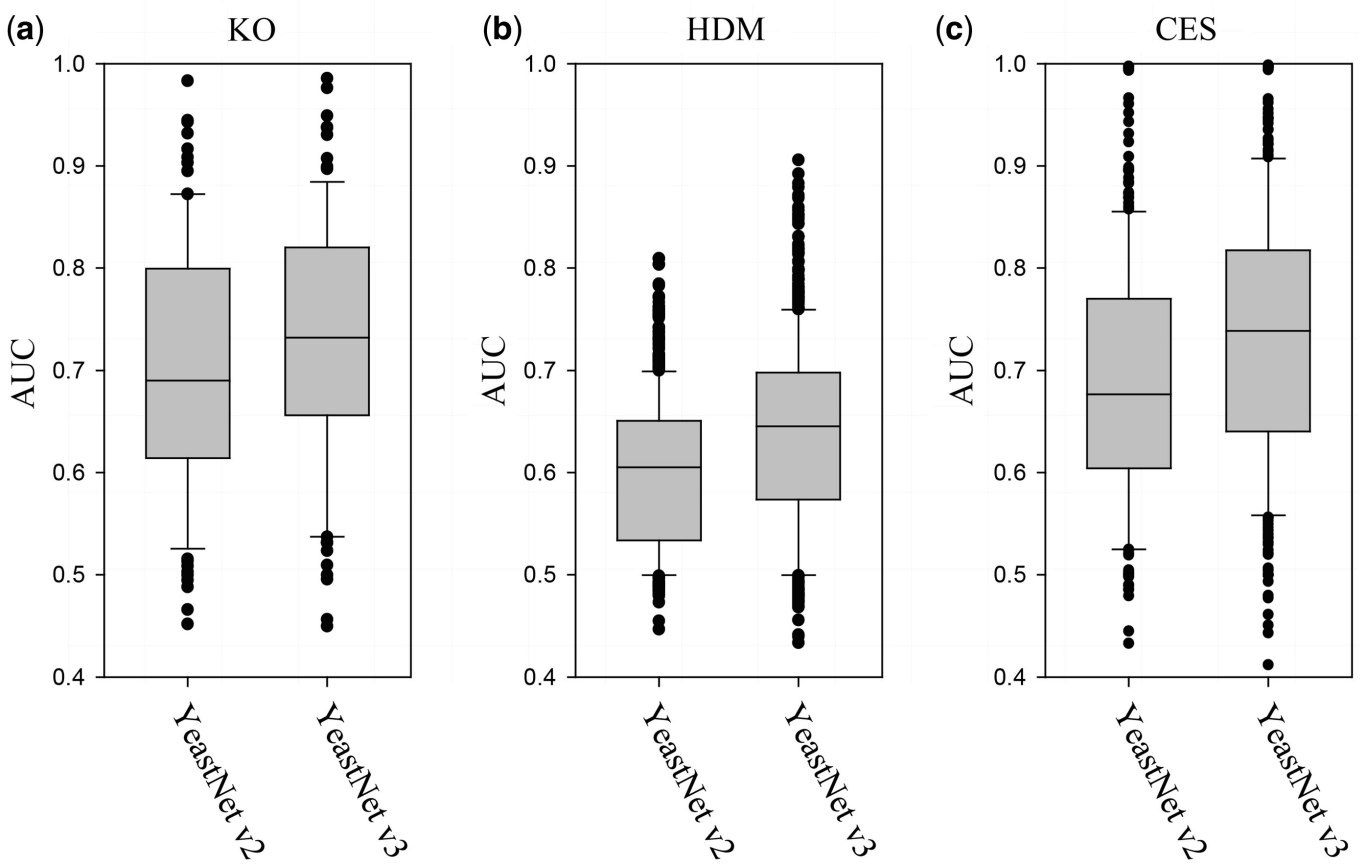
Box-and-whisker plots summarize the predictive power of networks for various phenotype data sets: (**a**) 100 knockout phenotypes (KO); (**b**) 586 high-dimensional morphology parameters (HDM); and (**c**) 88 types of chemical/environmental sensitivities (CES). The predictive power of the phenotypes was measured by an ROC curve analysis and summarized as area under the curve (AUC). AUC scores present how well a network recovers the connectivity among genes for a given phenotype, where an AUC of 0.5 indicates a prediction based on chance and an AUC of 1 indicates a perfect prediction. In the given box-and-whisker plots, the boundaries of the box represent the first and third quartiles, the whiskers represent the 10th and 90th percentiles, and the black circles represent individual outliers.

The holy grail of genetics is to understand the genetic organization of phenotypes. Therefore, we have pursued the development of gene networks as a platform to dissect the genetics of complex phenotypes. The previous version of YeastNet was able to predict the loss-of-function phenotype (4). We tested whether the improved quality of the current version of the network was able to generate a more sensitive and cost-effective genetic prediction for diverse phenotypes. First, we compared YeastNet v3 with the previous version of YeastNet using KO phenotype data. We used the same set of 100 KO phenotypes collected from the literature that was used for the previous network study (4) and assessed the predictive power of the networks by a receiver operating characteristic (ROC) curve analysis. Based on the leave-one-out method of cross-validation, we measured the prioritizing power of every test gene for each phenotype by the network links among member genes in the network. The resultant ROC curves were summarized by area under the curve (AUC) scores, which are summarized in Figure 2 for 100 tested KO phenotypes. We found that YeastNet v3 shows significant improvements in the prediction of the 100 KO phenotypes compared with the previous network (Figure 2a, *P* < 1.17 × 10^−^^9^, Wilcoxon signed rank sum test)

Most, if not all, phenotypes are quantitative such that the loss of a single gene does not shut down an entire system but provides some extent of phenotypic influence. The degree of phenotypic influence varies by gene and follows an approximately normal distribution. We used genome-wide high-dimensional morphology (HDM) profile data (9) and chemical/environmental sensitivity (CES) profile data (10) to test the predictive power of the network for quantitative phenotypes. There were 501 different HDM parameters that were profiled for 4718 genes with KO mutants. We generated 1002 test gene sets using genes with extreme morphological parameter values from both sides of the distribution (with a *P*-value threshold of 10^−^^4^). Among these test gene sets, we used 586 sets that each contained no less than five member genes to assess the predictive power of the network. From the CES database, we selected CES phenotypes that had no less than five sensitive genes with a *P*-value of 10^−^^7^ after five generations, for a total of 88 gene sets. For both quantitative phenotype data sets, we observed a significant improvement in YeastNet v3 compared with the previous network (Figure 2b and c, *P* < 2.2 × 10^−^^16^ for both HDM and CES, Wilcoxon signed rank sum test). From these results, we conclude that the improved network quality has enhanced the effectiveness of YeastNet v3 for predictive genetics research.

## WEB-BASED SERVICES FOR YeastNet v3

### Network-guided novel hypothesis generation

**Figure 3.**
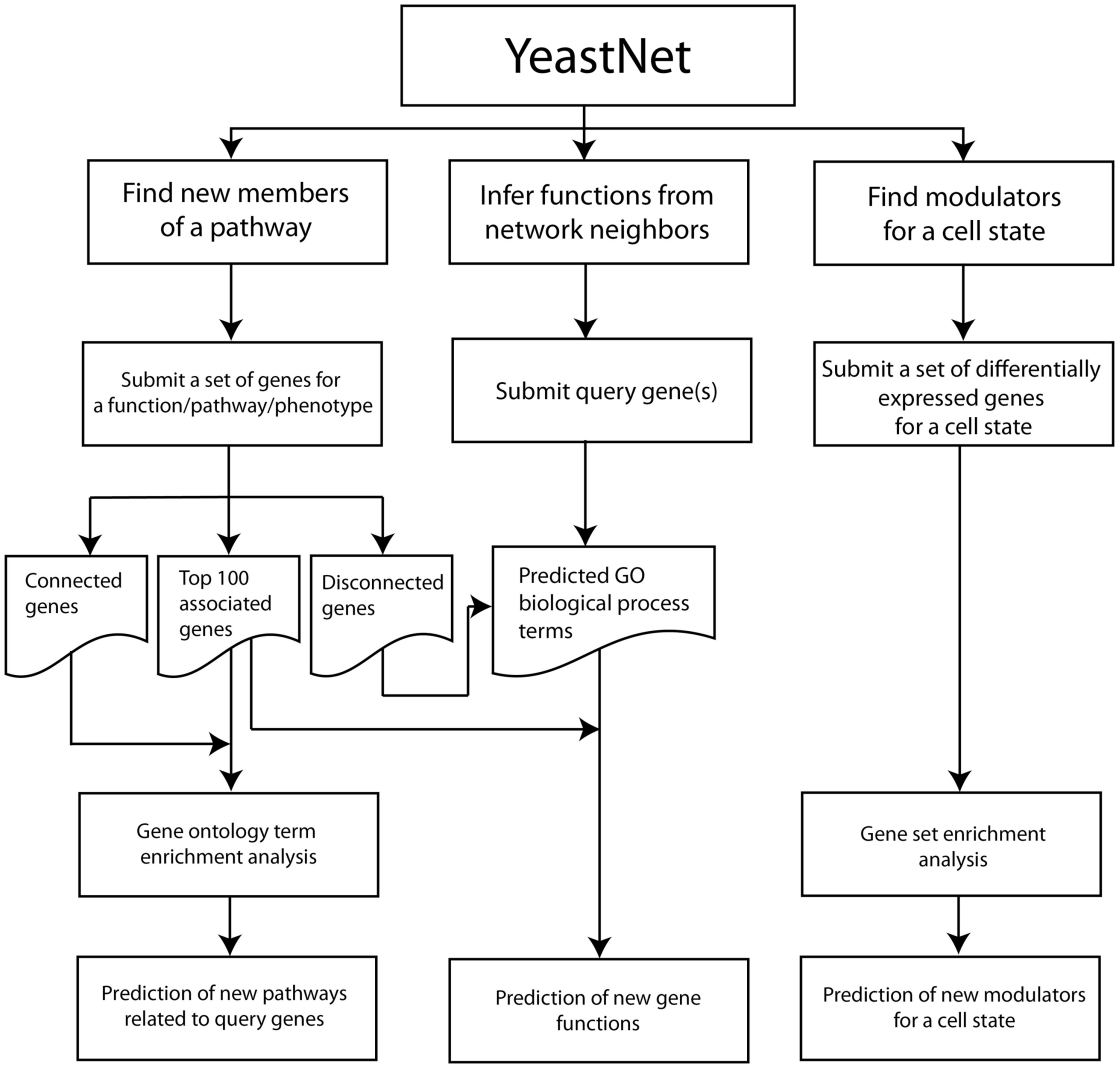
A schematic figure of the three options for network-guided hypothesis generation that are implemented in the YeastNet v3 web server.

The principle of guilt-by-association has been an effective method to generate novel hypothesis based on molecular networks. The previous version of YeastNet was used to successfully identify novel ribosomal biogenesis genes (11) and shmoo localized proteins (12). Both the quality and usability of a network are important to promote network-guided predictive genetics among experimental biologists. Therefore, we completely re-implemented the web interface for YeastNet v3 to provide three options for hypothesis generation under ‘network search’ (Figure 3): (i) find new members of a pathway; (ii) infer functions from network neighbors; and (iii) find modulators for a cell state.

From the ‘Find new members of a pathway’ option, users can predict novel candidate genes for a query functional concept (e.g. function, pathway or phenotype) based on the network associations of the user-provided genes that are known to be related to the query functional concept. Users can also visualize a network for the user-provided genes and their neighbors on YeastNet v3 using Cytoscape web (13). From the ‘Infer functions from network neighbors’ option, users can also predict novel functions for uncharacterized genes. If a user submits a query gene, then YeastNet looks up its network neighbors, collects their annotated Gene Ontology biological process terms and lists the 30 most enriched annotations.

YeastNet also provides a tool to predict modulators for a cell state. Yeast cells change their physiological state in response to harsh environments or chemical treatments. Many genetic modulators are involved in the transition between cell states. Although modulators are often transcriptionally activated, many modulators do not significantly alter their transcription levels during cell state modulation. Therefore, the expression profile may be insufficient to identify the genetic modulators for a cell state. Our hypothesis is that the cell state modulators are functionally coupled in YeastNet. Therefore, network links could identify modulators with no transcriptional change by their connections to genes with transcriptional changes. For the ‘Find modulators for a cell state’ option, the user submits a set of differentially expressed genes (DEGs) for a cell state. YeastNet v3 predefines gene sets for each gene in the network with their network neighbors such that the name of the gene set was assigned by the central hub gene. For the given query DEGs, the network server calculates the significance of the association between the DEGs and each gene set by hypergeometric probability. If a gene set is associated with the DEGs, its central hub gene is a candidate modulator for the query cell state. For example, we submitted 66 DEGs upon the treatment of an antifungal drug, sampangine (14). Because sampangine perturbs heme metabolism, the treatment of the drug shows a similar transcriptional response to hypoxia. The results showed that many genes that are required for anaerobic growth were highly ranked. However, many of the top candidates did not show a transcriptional response to sampangine, but did show strong associations to the DEGs by network links. For example, FLR1, a multidrug transporter, which might modulate drug response state was highly ranked (sixth) by YeastNet, but did not show a transcriptional response. Therefore, YeastNet provides a complement to DEG analysis to find cell state modulators.

### Availability of data-specific functional link information

A researcher may want to investigate alternative methods to integrate networks or perform in-depth analysis for each type of functional association between genes. To meet these potential demands, YeastNet v3 releases network edge information for data-specific networks in addition to the integrated network. This information can be downloaded from the ‘Network-download’ page. YeastNet v3 provides networks for all individual data sets (50 sets for CX, 10 sets for GT and 12 sets for HT), which include a total of ∼2 million edges.

## CONCLUSIONS

YeastNet v3 is a freely available database that provides a comprehensive cofunctional gene network for *S. cerevisiae*. This version of YeastNet has been substantially improved from previous versions by including novel linkage discovery algorithms, additional input data and versatile web-based analysis tools. YeastNet users will be able to generate diverse biological hypotheses with higher accuracy and sensitivity. We also expect that YeastNet v3 will improve other related networks and network construction tools using orthology-based links from YeastNet (15). We are confident that YeastNet v3 will provide a significant resource for predictive genetics and continue to facilitate new challenges in systems biology.

## SUPPLEMENTARY DATA

Supplementary Data are available at NAR Online.

## FUNDING

National Research Foundation of Korea [2010-0017649, 2012M3A9B4028641, 2012M3A9C7050151] and the Next-Generation BioGreen 21 Program [SSAC, PJ009029 to I.L.]. Funding for open access charge: National Research Grant.

*Conflict of interest statement*. None declared.
